# Alginate from *Ericaria crinita* Possesses Antioxidant Activity and Attenuates Systemic Inflammation via Downregulation of Pro-Inflammatory Cytokines

**DOI:** 10.3390/md22110482

**Published:** 2024-10-25

**Authors:** Paolina Lukova, Vesela Kokova, Alexandra Baldzhieva, Marianna Murdjeva, Plamen Katsarov, Cédric Delattre, Elisaveta Apostolova

**Affiliations:** 1 Department of Pharmacognosy and Pharmaceutical Chemistry, Faculty of Pharmacy, Medical University-Plovdiv, Vasil Aprilov Str. 15A, 4002 Plovdiv, Bulgaria; 2 Department of Pharmacology, Toxicology, and Pharmacotherapy, Faculty of Pharmacy, Medical University-Plovdiv, Vasil Aprilov Str. 15A, 4002 Plovdiv, Bulgaria; 3 Department of Medical Microbiology and Immunology “Prof. Dr. Elissay Yanev”, Faculty of Medicine, Medical University-Plovdiv, Vasil Aprilov Str. 15A, 4002 Plovdiv, Bulgaria; 4 Research Institute at Medical University-Plovdiv, Vasil Aprilov Str. 15A, 4002 Plovdiv, Bulgaria; 5 Department of Pharmaceutical Sciences, Faculty of Pharmacy, Medical University-Plovdiv, Vasil Aprilov Str. 15A, 4002 Plovdiv, Bulgaria; 6 Clermont Auvergne INP, CNRS, Institut Pascal, Université Clermont Auvergne, 63000 Clermont-Ferrand, France; 7 Institut Universitaire de France (IUF), 1 rue Descartes, 75005 Paris, France

**Keywords:** alginate, *Cystoseira crinita*, *Ericaria crinita*, antioxidant effect, anti-inflammatory effect, cytokines, IL-1β, TNF-α, Il-6

## Abstract

Alginates are anionic polysaccharides present in the cell walls of brown seaweeds. Various biological activities of alginate and its derivatives have been described. In this study, we assessed the potential of alginate obtained from *Ericaria crinita* (formerly *Cystoseira crinita*) to scavenge free radicals and function as a ferric ion reductor. The anti-inflammatory effect on the serum levels of TNF-α, IL-1β, IL-6, and IL-10 of rats with LPS-induced systemic inflammation after 14 days of treatment was also examined. *Ericaria crinita* alginate showed antioxidant activities of IC_50_ = 505 µg/mL (DPPH) and OD_700_ > 2 (ferric reducing power). A significant decrease in serum levels of IL-1β was observed only in animals treated with the polysaccharide at a dose of 100 mg/kg bw. Both doses of *E. crinita* alginate (25 and 100 mg/kg bw) significantly reduced the serum concentrations of pro-inflammatory cytokines TNF-α and IL-6, but no statistical significance was observed in the levels of the anti-inflammatory cytokine IL-10. Our findings show the potential of *E. crinita* alginate to act as an antioxidant and anti-inflammatory agent. It is likely that the exhibited antioxidant ability of the polysaccharide contributes to its antiphlogistic effects. More in-depth studies are needed to fully understand the specific mechanisms and the molecular pathways involved in these activities.

## 1. Introduction

Inflammation is a key component of the immune system of the body and an essential mechanism to survive against pathogens and chemical or mechanical stress. While normally a tightly controlled response, inflammation must be monitored to prevent its spreading and the cause of irreparable damage. Excessive production of pro-inflammatory cytokines, chemokines, and reactive oxygen species, primarily secreted by macrophages and other immune cells as lymphocytes, can cause severe impairment and contribute to the pathogenesis of different inflammatory diseases [1].

Lipopolysaccharide (LPS) is a potent endotoxin that is a component of the outer membrane of Gram-negative bacteria, which plays a major role in the pathogenesis of inflammatory diseases [2,3]. LPS stimulates the activity of immune cells and in macrophages causes excessive release of pro-inflammatory mediators through activation of nuclear factor-kappa B (NF-κB) and mitogen-activated protein kinase (MAPK) signaling pathways [4]. Such critical mediators produced by LPS-stimulated macrophages are prostaglandins and NO, which are generated following the activation of cyclooxygenase-2 (COX-2) and nitric oxide synthases (NOS), respectively [5]. Many inflammatory cytokines, including tumor necrosis factor (TNF)-α, interleukin (IL)-6, and IL-1β, can be secreted by LPS-activated mononuclear phagocytes (monocytes and macrophages) and other types of cells [6]. The increase of these inflammatory mediators levels is closely associated with the development of inflammation-related diseases [7]. Therefore, inhibition of these inflammatory cytokines is considered to be an effective strategy for anti-inflammatory therapy.

Reactive oxygen species (ROS) are a group of short-lived, highly reactive molecules that are involved in numerous physiological processes like cell maturation, signaling, immune defense, and cytotoxicity against pathogens [8,9]. However, the overproduction of ROS is recognized as causing cellular abnormalities such as DNA alteration, lipid peroxidation, protein damage, and enzyme deactivation, which contribute to pathological conditions such as autoimmune disorders, cardiovascular diseases, atherosclerosis, rheumatoid arthritis, diabetes, and tumorigenesis [10,11]. Generally, antioxidants are defined as synthetic or natural substances that at low concentrations can prevent, inhibit, or delay the deterioration of oxidizable substrates [12]. The European Food Safety Authority (EFSA) assesses the scientific evidence regarding the use, benefits, and safety of antioxidants in food products and supplements [13].

While numerous synthesized chemicals effectively scavenge free radicals, they often have significant side effects. Moreover, available anti-inflammatory agents, such as nonsteroidal anti-inflammatory drugs and steroids, cause serious adverse effects with long-term use. Therefore, developing alternative treatments against inflammation and oxidative stress that are effective and less toxic remains an ongoing challenge. Natural products play an essential role in the process of new drug discovery and development. Nowadays, significant focus has been placed on the preparation and modification of polysaccharides, especially marine-derived polysaccharides such as fucoidans, alginates, laminarins, ulvans, agar, and chitosan [14,15,16].

Alginates are biopolymers commercially produced from brown marine algae; however, bacterial alginates from some *Pseudomonas* sp. and *Azotobacter* sp. are also used. Structurally alginates are composed of β-D-mannuronic acid (M) and its C-5 epimer, α-L-guluronic acid (G), linked by β-(1,4) bonds. The mannuronic and guluronic residues are arranged in homopolymeric (MM, GG) or heteropolymeric block structures (MG, GM) [17,18]. Alginates’ MG block ratio, molecular weight, and molecular conformations block structure significantly influence their biological and physicochemical properties. Alginates have numerous proven properties, such as being non-toxic, water-soluble, biodegradable, biocompatible, film-forming, gelling, thickening, and anti-allergic [19,20]. Moreover, alginates are also known for their anti-inflammatory, antibacterial, antioxidant, prebiotic, mucoprotective, antidiabetic, and anti-obesity properties [17,18,19].

In this study, we aimed to achieve the following: (a) to examine the anti-inflammatory effect of the previously extracted and characterized alginate derived from the brown algae *Ericaria crinita* (*E. crinita*) (formerly *Cystoseira crinita*) [17] on the serum levels of TNF-α, IL-1β, IL-6, and IL-10 of rats with LPS-induced systemic inflammation after 14 days of treatment, (b) to evaluate the antioxidant capacity of *E. crinita* alginate to scavenge free radicals and to reduce ferric ions by donating an electron, and (c) to compare the obtained results with the activities of alginate structures isolated from other marine algae. Therefore, the performed analyses will highlight the importance of utilizing natural antioxidants and anti-inflammatory substances of marine origin, with a particular emphasis on alginate derived from *E. crinita.* By advancing the understanding of marine polysaccharides’ therapeutic potential, this research will contribute to the development of safer, more sustainable, and effective alternatives to synthetic compounds in treating oxidative stress and inflammation, improving health outcomes.

## 2. Results

### 2.1. Changes in Serum Levels of Pro-Inflammatory Cytokines (IL-1β, TNF-α, and IL-6)

**Figure 1:**
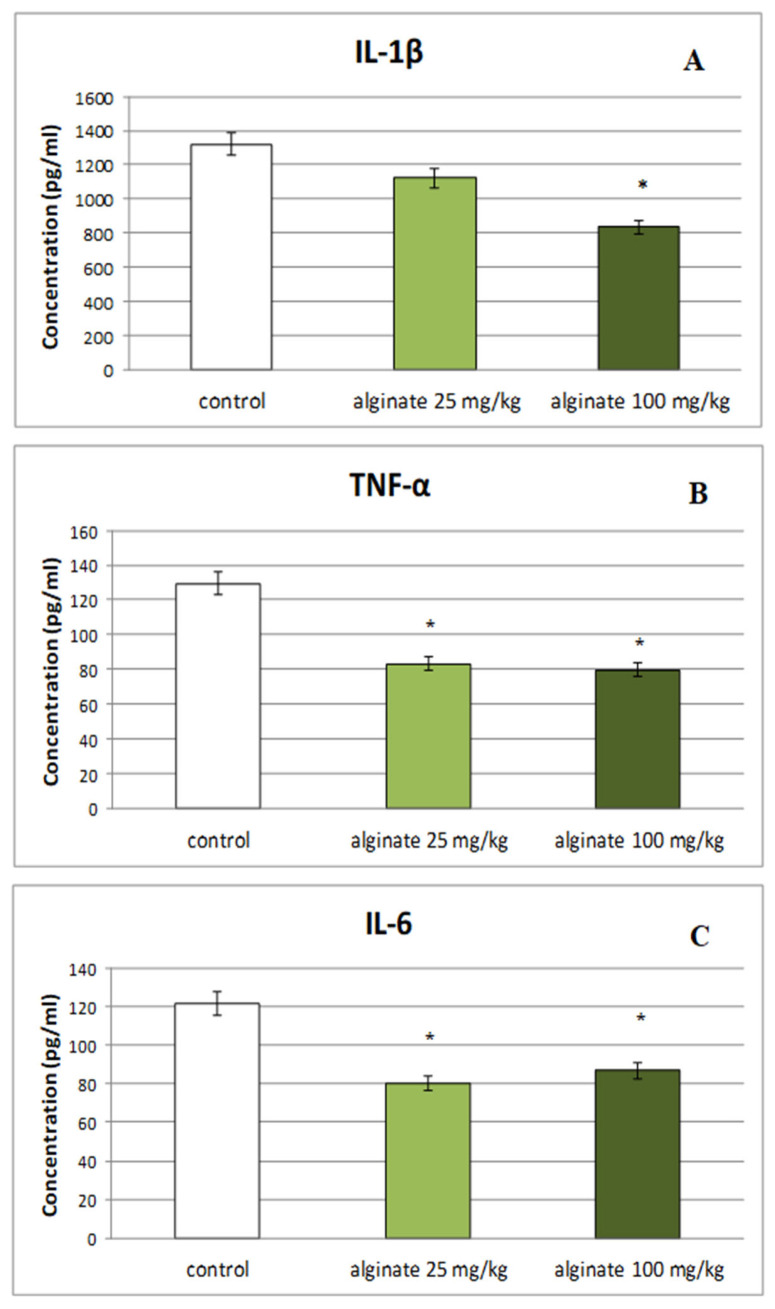
Effect of subchronic treatment with alginate from *E. crinita* (25 and 100 mg/kg bw) on serum levels of the pro-inflammatory cytokines IL-1β (panel (**A**)), TNF-α (panel (**B**)), and IL-6 (panel (**C**)) in LPS-induced systemic inflammation in rats. * *p* < 0.05 vs. same cytokine controls.

A significant decrease in serum levels of IL-1β was observed only in animals treated with the higher dose of alginate from *E. crinita* (100 mg/kg bw) in comparison to controls (835.13 ± 70.22 vs. 1318.97 ± 171.14; *p* < 0.05; Figure 1A). The reducing effect was also observed in TNF-α serum levels of rats treated with alginate isolated from *E. crinita* at a dose of 25 mg/kg bw (83.31.48 ± 8.37 vs. 129.33 ± 13.81; *p* < 0.05) and 100 mg/kg bw (79.67 ± 7.36 vs. 129.33 ± 13.81; *p* < 0.05) when compared with the control group (Figure 1B). As shown in Figure 1C, the levels of IL-6 in the serum of rats treated with both doses of *E. crinita* alginate were significantly decreased (80.29 ± 7.25 for the lower dose and 86.89 ± 7.82 for the higher dose vs. 121.08 ± 11.16; *p* < 0.05) in comparison to controls.

### 2.2. Changes in Serum Levels of Anti-Inflammatory Cytokine IL-10

**Figure 2:**
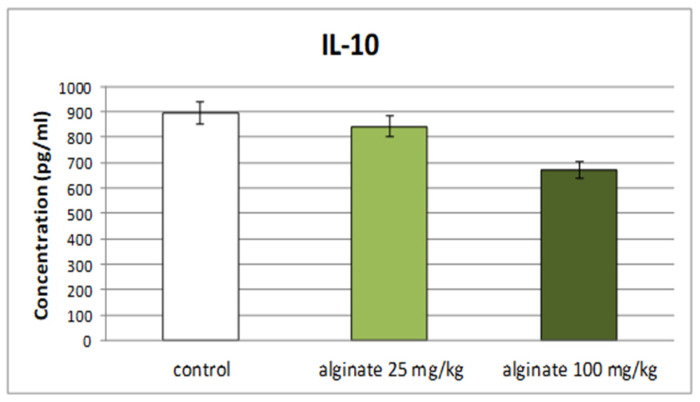
Effect of subchronic treatment with alginate from *E. crinita* (25 and 100 mg/kg bw) on serum levels of the anti-inflammatory cytokine IL-10 in rats with LPS-induced systemic inflammation.

Subchronic treatment of rats with *E. crinita* alginate at doses of 25 mg/kg and 100 mg/kg bw did not significantly change serum levels of the anti-inflammatory cytokine IL-10 in a model of LPS-induced systemic inflammation in comparison to controls (Figure 2).

### 2.3. 2,2-Diphenyl-1-Picryl-Hydrazyl-Hydrate (DPPH) Radical Scavenging Effect

**Figure 3:**
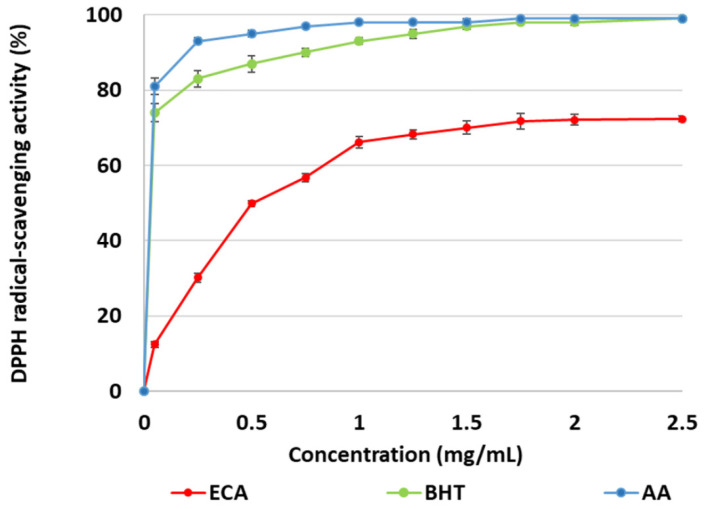
DPPH radical scavenging activity of *E. crinita* alginate (ECA), butylated hydroxytoluene (BHT), and ascorbic acid (AA).

As shown in Figure 3, the sample of *Ericaria crinita* alginate (ECA) was able to produce a stable form of DPPH in a dose-dependent manner. The inhibitory effect of ECA varied from 12.45% to 72.36% at concentrations of 0.05–2.5 mg/mL. Furthermore, in the range from 1.75 to 2.5 mg/mL, no remarkable augmentation of the antioxidant activity of ECA (from 71.78% to 72.36%) was established. An IC_50_ value of 505 µg/mL was calculated for ECA, which was lower than those of the two commercial antioxidants butylated hydroxytoluene (BHT) (IC_50_ = 5.5 µg/mL) and ascorbic acid (AA) (IC_50_ = 4.1 µg/mL).

### 2.4. Ferric Reducing Power

**Figure 4:**
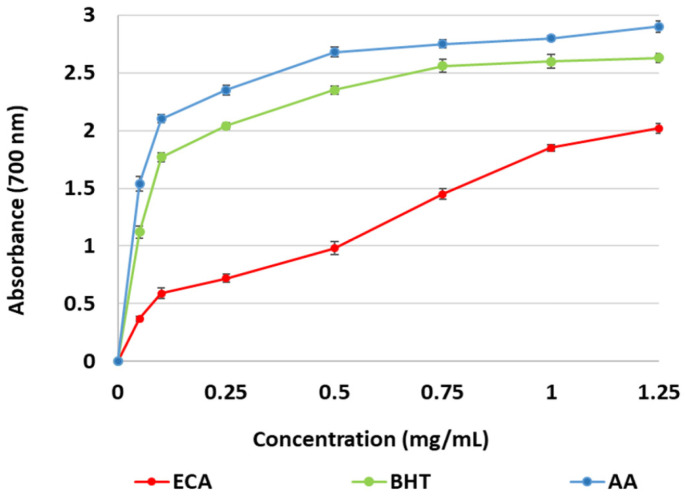
Ferric reducing power of *E. crinita* alginate (ECA), butylated hydroxytoluene (BHT), and ascorbic acid (AA).

Figure 4 illustrates the capacity of ECA to facilitate the reduction of ferric to ferrous ions in a redox-linked colorimetric reaction. The OD_700_ of ECA exhibited a considerable range, from 0.37 to 2.02, across a concentration spectrum of 0.05 to 1.25 mg/mL. Hence, the most pronounced rise in activity, almost 50%, was noted between 0.5–0.75 mg/mL, respectively from 0.98 to 1.45. However, BHT and AA demonstrated higher reducing capacity in the tested concentrations (Figure 4).

## 3. Discussion

Alginates and their oligosaccharides have attractive pharmaceutical properties that exert many beneficial biological activities [19,21]. However, little is known about the anti-inflammatory effect of alginate isolated from *E. crinita.* Previously, we reported decreased levels of TNF-α, IL-6, and IL-1β after a single dose of *E. crinita* alginate [17]. In the current study, we found that *E. crinita* alginate at a dose of 25 and 100 mg/kg bw could ameliorate LPS-induced systemic inflammation after 14 days of treatment. We discovered that alginate from *E. crinita* could reduce serum levels of the pro-inflammatory cytokines IL-1β, TNF-α, and IL-6. These data confirm that alginate protects and possesses anti-inflammatory activity.

Our results are supported by the in vivo and in vitro findings of many other authors. Alginate isolated from *E. maxima* stipes attenuates particulate matter-induced inflammation in lung macrophages and downregulated the production of pro-inflammatory cytokines TNF-α, IL-6, and IL-1β [22]. The anti-inflammatory effects of oral and intraperitoneal administrated alginate gels in experimental models of ulcerative colitis and glomerulonephritis have been reported [23]. Low-viscosity sodium alginate (LVA), purified from *Macrocystis pyrifera* (concentration of 0.5% (*w*/*v*), administrated orally in drinking water for 1 week) suppressed the progression of colonic inflammatory lesions and decreased the serum and colonic mucosal levels of IL-6, TNF-α, leukotriene B4 (LTB4), and prostaglandin E2 (PGE2), in a model of acute colitis in rats [24]. Furthermore, its 6-week application reduced these serum levels, as well as colonic mucosal secretion of IL-6, TNF-α, LTB4, and the colonic damage score, in a model of chronic ulcerative colitis in rats [25]. The same LVA (dose 50 mg/kg bw, administrated i.p.) decreased proteinuria and serum creatinine, and inhibited antibody production and the glomerular deposition of immune complexes, as well as the development of glomerular lesions, in a model of immune complex glomerulonephritis in rats [26]. In another in vitro study, alginic acid from *Macrocystis pyrifera* with an approximate composition of 61% mannuronic and 39% guluronic acid, inhibited IL-1β and TNF-α production and mRNA expression but not IL-6 and IL-8 secretion. The authors suggested that alginic acid has different regulatory effects, including mast cell stabilization and inhibition of NF-kB, which might explain its anti-inflammatory properties [27]. Evidence of a local anti-inflammatory effect of sodium alginate and its compounds has been found in several studies [28,29,30,31].

Nevertheless, some research data indicate that alginate may have pro-inflammatory properties. For instance, sodium alginate with a molecular weight of 9500 kDa and an M/G ratio of 1.96 activated RAW264.7 macrophages, leading to increased levels of the pro-inflammatory cytokines TNF-α, IL-1β, IL-6, and IL-12 and triggered innate immunity via NF-κB activation [32]. Another study reported that high mannuronic acid-containing alginate (HMA) derived from *Macrocystis pyrifera* has an immunostimulatory effect, increases the number of peritoneal macrophages in mice, and promotes phagocytosis, secretion, and tumoricidal activity of macrophages through the production of cytokines and cytotoxic molecules such as TNF-α, NO, and H_2_O_2_ [33]. Additionally, alginate from *Ascophyllum nodosum* with high M- and MG-blocks was significantly more effective in inducing TNF-α IL-1, and IL-6 production than alginate with high G-blocks from *Laminaria digitata* [34]. Mannoglucan was also shown to have TNF-α-like antitumor activity [35]. These findings contrast with the anti-inflammatory effects of alginate observed in our studies. The first reason may be that these experiments were conducted in vitro whereas ours were performed in vivo. Secondly, different factors may influence the biological activities of alginates and alginate oligosaccharides (AOS), including their structure, molecular weight, M and G content (M/G ratio), and spatial conformation of molecules. Other factors, such as molecular weight, algal source, and extraction methods, should also be considered. This also applies to the immunogenicity of alginate, which can vary depending on its chemical composition. A higher proportion of mannuronic acid in alginate, such as the structure in the last cited studies, could enhance its immunostimulatory effects. For example, the M/G ratio of the examined alginate in Yang and Jones’ research [32] was 1.96. The content of β-D-mannuronic acid in our *E. crinita* alginate was lower (M/G ratio 1.018). Likewise, the anti-inflammatory properties of AOS are dependent on the M and G monomer content. M−block AOS were found to increase the production of TNF-α, RANTES, and granulocyte-colony stimulating factor (G-CSF) from RAW macrophages as compared to G-block AOS [36]. Thus, alginates rich in mannuronic acid appear to stimulate pro-inflammatory cytokine production and activate the innate immune response via the NF-κB pathway. On the other hand, G-block AOS were reported to reduce the secretion of ROS, NO, PGE2, IL-1β, TNF-α and IL-6, and the expression of COX-2 after LPS activation [37]. It was proposed that G-block AOS can prevent the binding of LPS to Toll-like receptor 4 (TLR4), averting downstream NF-κB signaling [38].

Monocytes and macrophages respond to bacterial LPS and activate several host defense functions through the secretion of many inflammatory mediators. When activated with a specific source of LPS, monocytes exhibit rapid expression of mRNA for TNF-a, IL-1β, and IL-8, which is followed by IL-6 [39]. TNF-α can cause inflammation and apoptosis and can mediate the release of other cytokines, such as IL-6 and IL-8 by stimulating macrophages [40]. It is known that IL-1β is a potent pro-inflammatory cytokine that leads to vasodilatation, promotes the attraction of granulocytes to the site of inflammation, and induces prostaglandin production during an acute inflammation [41]. IL-1β also participates in chronic inflammation and stimulates other cytokine secretion by Th17 cells, promoting the development of chronic inflammatory diseases [42]. Furthermore, sensitive to IL-1β is a subtype of Th1 cells that express CD161, functionally related to Th17 cells [43]. Besides the pro-inflammatory activity, IL-1β plays a key role in the innate immune response. IL-1β has been suggested to link innate and acquired immunity through its additional effects on T cells [44]. IL-6 is a pleiotropic pro-inflammatory cytokine that participates actively in inflammatory and immunomodulatory mechanisms. Its production is mainly activated by IL-1β and TNFα, although there are also other ways to promote its secretion such as Toll-like receptor activation (TLRs), stress response, prostaglandins, adipokines, and other cytokines [45]. IL-6 induces an increase in the concentrations of various acute phase proteins such as C-reactive protein (CRP), serum amyloid A, fibrinogen, haptoglobin, and α1-antichymotrypsin, whereas it inhibits the production of albumin. These changes in acute-phase protein levels are used to evaluate the severity of inflammation in routine clinical laboratory tests. IL-6 not only evokes acute phase reactions but also leads to the development of specific cellular and humoral immune responses and plays an important role in acquired immunity by end-stage B-cell differentiation, stimulation of antibody secretion, and T-cell activation. Thus, IL-6 is an important mediator for the transition from acute to chronic stage of inflammation and performs an important function in the linking of innate to acquired immune response. Dysregulated continual synthesis of IL-6 is associated with the development of autoimmune and chronic inflammatory diseases [46,47,48]. Our data suggest that alginate could reduce inflammation induced by LPS. Our study found that TNF-α, IL-6, and IL-1β levels were decreased by *E. crinita* alginate. Alginate could ameliorate LPS-induced systemic inflammation by modulating serum levels of cytokines related to inflammation.

Cytokines and reactive oxygen species are closely interconnected [49]. Free radicals play a crucial role in triggering and maintaining inflammatory processes, so neutralizing them with antioxidants and radical scavengers can help reduce inflammation [50]. One study reported that alginic acid, derived from the brown algae *Sargassum wightii*, showed anti-inflammatory and antioxidant effects in rats with arthritis induced by complete Freund’s adjuvant when administered orally at a dose of 100 mg/kg. The treatment led to a reduction in paw edema, decreased COX-2 and 5-LOX activities, and less neutrophil infiltration. Additionally, alginic acid decreased lipid peroxidation by enhancing the cellular antioxidant defense system, increasing the activity of antioxidant enzymes, and boosting reduced glutathione levels [51]. Furthermore, alginate inhibited TNF-α-induced intercellular adhesion molecule-1 expression, nitric oxide production, and hydrogen peroxide levels [52], suggesting that the antioxidant properties of alginate contribute to its anti-inflammatory effects.

Studies on the antioxidant activity of alginates demonstrated their in vitro ability to scavenge free hydroxyl and superoxide radicals, along with acting as a chelator/reductor of Fe ions and bleaching of β-carotene [53]. Several factors influence the antioxidant properties of alginates, including molecular weight, concentration, pretreatment techniques, period of collection, and in the case of alginate oligosaccharides, the method of depolymerization. Generally, the reported antioxidant effect was concentration- and time-dependent, and inversely proportional to alginate molecular weight [15,54]. Low molecular weight (LWM) alginates have better antioxidant ability compared to high molecular weight ones. It is supposed that this could be due to the presence of additional functional groups in the LWM alginates [53]. Moreover, polysaccharides with higher molecular weight exhibit a denser structure, leading to stronger intramolecular hydrogen linkages, which limit the accessibility of hydrogen groups [54]. This claim has been proven by several authors [18,54], including the results from our study for *E. crinita* alginate. Benslima et al. [54] isolated four alginate polymers from *Cystoseira schiffneri* collected in different months of the year with Mw ranging from 1.23 × 10^6^ to 4.49 × 10^3^ g/mol. The alginates with higher Mw (up to 2.33 × 10^5^ g/mol) exhibited a lower DPPH radical scavenging effect (IC_50_ > 1500 µg/mL), while the alginate with Mw = 4.49 × 10^3^ g/mol had an IC_50_ value of 190 µg/mL. Similarly, Borazjani et al. [55] reported the DPPH scavenging activity of four alginates obtained from the brown algae *Sargassum angustifolium* using different pretreatment agents. The enzyme-treated alginates resulted in the lowest Mw (357 × 10^3^ g/mol) and, respectively, the highest antioxidant properties (more than 66% of inhibition) (Table 1). The same inversely proportional relationship between molecular weight and DPPH antioxidant activity was observed when comparing the scavenging effect of the obtained alginate from *E. crinita* with those of other brown species: *Cystoseira compressa* [18], *Cystoseira schiffneri* [54], and *Sargassum angustifolium* [55] (Table 1). Contrary to the above-mentioned comparisons, Sellimi et al. [56] reported the highest value for the DPPH scavenging effect (IC_50_~150 µg/mL) of *Cystoseira barbata* alginate, which had a higher value of Mw (2.99 × 10^5^ g/mol). Nonetheless, Mw appears to be the major factor in the control of alginates’ DPPH antioxidant effect.

Moreover, some authors claim that the antioxidant activity of alginates is independent of the relative content of mannuronate and guluronate, considering that the only distinction between pM and pG is the orientation of the C5 carboxyl groups [21]. However, the study of Nouha Sari-Chmayssem et al. [57] demonstrated 25% higher hydroxyl radical scavenging activity for the homopolymeric polyguluronate fraction compared to the homopolymeric polymanuronate fraction with slightly similar molecular weight (Table 1). These observations could be attributed to the diaxial linkage in guluronate blocks, which may result in a restricted rotation around the glycosidic linkage. The enhanced flexibility of guluronate blocks may be responsible for the rigid and extended configuration of the alginate chains, which could affect the accessibility of the sodium alginate hydroxyl groups [57].

Allegedly, the ferric reducing power of polysaccharides was found to be related to their molecular weight and the number of hydroxyl and carboxyl groups present in the uronic acids [18,54]. *E. crinita* alginate at a concentration of 0.5 mg/mL showed values for ferric reducing antioxidant activity (OD_700_ = 0.98) comparable to those reported for alginate extracted from *Cystoseira compressa* (OD_700_~1) with similar uronic acid content but higher Mw [18]. Moreover, alginate from *C. barbata* with higher Mw and higher uronic acid exhibited lower ferric-reducing capacity (OD_700_~0.7) [56] (Table 1). Similarly to the DPPH results, Borazjani et al. [55] reported the highest reducing ability for *Sargassum angustifolium* alginate fraction, which had the lowest Mw. Nevertheless, contrary findings have also been reported by other researchers. Regarding the results from Benslima et al. [54], the reducing capacity of *Cystoseira schiffneri* alginate was not found to be correlated with either their uronic acid amount or their molecular weight. It may be necessary to consider additional factors, such as the polyphenol content retained in alginates or other structural characteristics [54,58].

The antioxidant and anti-inflammatory activities of alginate have significant implications for health, industry, and the environment. By further unveiling its mechanisms and applications, alginate could play a significant role in various health fields. The current study, for example, suggests the potential use of polysaccharides for the treatment and prevention of inflammatory diseases. Moreover, by neutralizing free radicals, alginate can reduce cellular damage and potentially lower the risk of chronic disorders. The ability of alginate to modulate inflammatory pathways can help manage these conditions, offering therapeutic approaches for reducing severe symptoms and improving quality of life. Furthermore, alginate’s antioxidant properties can be utilized in food preservation, extending food product shelf life by preventing oxidation and improving its chemical stability. Due to their unique properties and valuable therapeutic effects, alginates are also widely exploited in pharmaceutical technology. As natural biodegradable materials, these polysaccharides are preferred excipients and drug carriers, whose antioxidant and anti-inflammatory properties can enhance the efficacy of certain active substances, particularly in treating diseases characterized by oxidative stress and inflammation.

## 4. Materials and Methods

### 4.1. Algae Material and Chemicals

Talluses of the brown algae *Ericaria crinita* (Duby) Molinari & Guiry were gathered from the Black Sea coast, Bulgaria, in July 2019. The taxonomy, species identification, and pretreatment of the algae were detailed in our earlier research [17]. Test substances used in the experiments were alginic acid sodium salt (Product No. 180947), lipopolysaccharides from *E. coli* O55:B5 (LPS), butylated hydroxytoluene, ascorbic acid, and 2 and 2,2-diphenyl-1-picryl-hydrazyl-hydrate. All the reagents were purchased from Sigma Aldrich. The tested alginates and LPS were dissolved in saline.

### 4.2. Animals

Male Wistar 4-month-old rats weighing 160–205 g were used. The animals were housed in standard laboratory conditions: temperature 22 °C ± 1 °C, humidity 45%, light/dark cycle of 12/12 h, with access to food and water ad libitum.

### 4.3. Extraction and Structural Characterization of Ericaria crinita Alginate

The procedure of the alginate extraction and its structural analysis were outlined in our prior study [17]. *E. crinita* sodium alginate was extracted using an alkaline solvent (3% Na_2_CO_3_, pH 11) for 2 h, at a temperature of 60 °C. The extraction yield was determined to be 20.18% based on the dry weight of the algae. The uronic acid and neutral sugar contents were estimated to be 50.14% and 19.66%, respectively. The purity of *E. crinita* alginate was evaluated based on the low percentage of sulfate groups (0.63%), proteins (less than 0.04%), and total polyphenols (less than 0.10%). The average molecular weight in mass (Mw) and average molecular weight in number (Mn) of *E. crinita* alginate were estimated using size-exclusion chromatography–multi-angle light scattering (SEC/MALS). The calculated Mn and Mw were found to be 5.29 × 10^4^ g/mol and 7.31 × 10^4^ g/mol, respectively. The polydispersity index of 1.38 indicated a narrow mass distribution, confirming the polymer’s homogeneity. The Fourier-transform infrared (FTIR) spectroscopy showed two characteristic peaks at 1090 and 1035 cm^−1^ corresponding to the mannuronic and guluronic acid. Further analysis of the chemical structure and block distribution of the alginate polymer was accomplished using proton nuclear magnetic resonance (^1^H NMR) spectroscopy. The results revealed typical 500 MHz-^1^H NMR signals attributed to repeating guluronic and mannuronic acid units. M/G ratio of 1.018 was estimated, indicating relatively equal levels of β-D-mannuronic and α-L-guluronic acids. Moreover, the homopolymorphic MM or GG regions were almost equivalent to the alternating blocks. The FTIR and ^1^H NMR spectra of the *E. crinita* alginate revealed no traces of fucoidan, as confirmed by the absence of characteristic signals from the methyl groups in the fucose and sulfate ester groups.

### 4.4. Detection of Pro-Inflammatory and Anti-Inflammatory Cytokines

The effect of multiple applications of alginate from *E. crinita* on serum cytokine levels was studied. Twenty-four male Wistar rats were randomly divided into three experimental groups, each consisting of eight animals, and treated intraperitoneally for 14 days as follows: 1st group (control)—treated with saline (0.1 mL/100 g bw); 2nd group (alginate 25 mg/kg)—treated with 25 mg/kg bw alginate from *E. crinita*; and 3rd group (alginate 100 mg/kg)—treated with 100 mg/kg bw alginate from *E. crinita*. Thirty minutes after the last application of the substances, a solution of LPS at a dose of 0.25 mg/kg was injected intraperitoneally. Four hours after the LPS challenge, blood samples were collected in test tubes for serum yield. They were transported immediately to the Department of Medical Microbiology and Immunology “Prof. Dr. Elissay Yanev” in an ice container.

The collected rat blood samples were promptly processed through centrifugation at 1000× *g* for 10 min at room temperature, using a Hermle Z36-HK centrifuge (HERMLE Labortechnik GmbH, Wehingen, Germany), situated in the Department of Medical Microbiology and Immunology “Prof. Dr. Elissay Yanev”. The centrifugation process separated the serum, which was then carefully removed from the monovettes using sterile techniques in order to avoid contamination. This serum was divided into smaller portions (aliquots), each ranging from 250 to 500 μL depending on the initial volume of the collected blood. This division was conducted to minimize the need for multiple freeze-thaw cycles, which could deteriorate the samples’ and the cytokines’ quality and stability. These aliquots were then placed in labeled cryovials and stored at −80 °C to preserve them for further examination.

The study focused on measuring the concentrations of four specific cytokines: Rat IL-1β, Rat IL-6, Rat IL-10, and Rat TNF-α in the serum samples. This was achieved using an enzyme-linked immunosorbent assay (ELISA) with pre-coated strip plates provided by Diaclone (CEDEX—Besançon, Franche-Comté, France). Those kits were selected due to their well-documented sensitivity, specificity, and compatibility with rat serum samples. The entire procedure was performed strictly following the manufacturer’s instructions to ensure accuracy and reliability.

Optical density measurements based on the linear relationship between light absorbance and particle concentration were taken at a wavelength of 450 nm, with an optional reference filter at 620 nm, using a Tecan Sunrise Microplate Reader (Tecan Austria GmbH, Salzburg, Austria) paired with the Magellan™ Data Analysis Standard v7.2 Software. To quantify the cytokine levels, a standard curve was generated using known concentrations of cytokine standards included in the ELISA kits. The concentrations of cytokines in the samples were then calculated using the linear equation derived from this standard curve. The results were expressed in picograms per milliliter (pg/mL), providing a precise measure of the cytokine levels in each serum sample.

### 4.5. DPPH Radical Scavenging Activity

The DPPH free radical scavenging activity of *E. crinita* alginate was conducted as per the method outlined by Kao and Chen [59], with slight modifications. Specifically, 0.5 mM DPPH in methanol (0.2 mL) was mixed with the alginate solution (1 mL) at concentrations ranging from 0.05 to 2.5 mg/mL. These mixtures were incubated at room temperature in darkness for 15 min. Subsequently, the reduction in absorbance at 517 nm was estimated using a UV-VIS spectrophotometer Evolution 300 (Thermo Fisher Scientific, Waltham, MA, USA). A blend of the polysaccharide solution and methanol served as a blank. The antioxidant efficacy was quantified as a percentage of inhibition, calculated using the equation:

$$\%\;Inhibition=\frac{A_{0}-A_{15}}{A_{0}}\times 100$$

where A_0_ denoted the blank sample absorbance at 0 min and A_15_ designated the polysaccharide solution absorbance after 15 min. Results were presented based on triplicate measurements with standard deviation (±SD), and the IC_50_ values were deduced from linear regression plots.

### 4.6. Ferric Reducing Power

The ferric reducing power was assessed using the method of Yildirim et al. [60]. A 0.3 mL volume of polysaccharide solution at different concentrations (0.05–1.25 mg/mL) was mixed with 1.25 mL phosphate buffer (0.2 M, pH 6.6) and 1.25 mL of 1% potassium ferricyanide. Following a 30 min incubation period at 50 °C, the mixture was treated with 10% 1.25 mL of trichloroacetic acid and subsequently subjected to centrifugation for 10 min at 3500 rpm. Then, 1.25 mL of the supernatant, 1.25 mL of distilled water, and 0.25 mL of 0.1% ferric chloride solution were mixed in a test tube. The ferric reducing power was estimated by measuring the increase in absorbance after 10 min at a wavelength of 700 nm. Blanks lacking ferric chloride were prepared for each concentration and BHA and AA were employed as standards. The values were determined through triplicate measurements with ±SD.

### 4.7. Statistical Analysis

Statistical evaluation was executed with SPSS 17.0 employing one-way ANOVA and Bonferroni post hoc tests. The normality of distribution was determined with the one-sample Kolmogorov–Smirnov test. The number of tested animals is given as *n*. Results are presented as arithmetic mean ± standard error of the mean (± SEM). *p*-value ≤ 0.05 was considered statistically significant.

## 5. Conclusions

Our study demonstrated that subchronic treatment with alginate isolated from *E. crinita* in doses of 25 mg/kg and 100 mg/kg bw could ameliorate LPS-induced systemic inflammation by reducing serum levels of pro-inflammatory cytokines IL-1β, TNF-α, and IL-6 in rats. The antioxidant activity of *E. crinita* alginate, as evidenced by DPPH radical scavenging and reducing power assays, showed a concentration-dependent increase. Although the obtained data on *E. crinita* alginate’s antioxidant and anti-inflammatory activities are encouraging, more in-depth studies are needed to fully understand the specific mechanisms and the molecular pathways involved in these processes. Future research may also focus on their multifunctional applications offering benefits beyond antioxidant and anti-inflammatory effects. The full potential of marine alginates can be harnessed to provide novel, effective, and sustainable therapeutic options for the treatment and prevention of various acute and chronic diseases.

The study has the following limitations: (1) the rats received intraperitoneal injections of alginate once a day for 14 days, so different treatment periods or application routes could result in different outcomes; (2) the results were obtained in rats and could not be extrapolated to humans without additional experiments; (3) the cytokines were evaluated 4 h after the LPS challenge, so changes in the time point of evaluation could have influenced the results; (4) the antioxidant properties of the compound were studied in vitro; however, there is no guarantee that the same activity will occur in vivo.

## Figures and Tables

**Table 1:** Compositional data of alginates extracted from different species of brown algae.

DPPH		OD_700_ (0.5 mg/mL)	Uronic Acids (%, *w*/*w*)	Mw (g/mol)	Period of Collection	Pretreatment	Algal Source	Ref.
IC_50_ (µg/mL)	% Inh. (1 mg/mL)							
505	66.2	0.98	50.14	7.31 × 10^4^	July	0.1 M HCl	*Ericaria cirnita* (*Cystoseira crinita*)	Current study
560	~70	~1.0	46.48	1 × 10^5^	February	0.1 M HCl	*Cystoseira* *compressa*	[18]
~150	~73	~0.7	58.1	2.99 × 10^5^	November	0.1 M HCl	*Cystoseira* *barbata*	[56]
>1500 >1500 >1500 190	~15 ~23 ~17 ~80	~2.0 ~0.3 ~1.0 ~2.8	47 53 66 62	1.23 × 10^6^ 1.16 × 10^5^ 2.33 × 10^5^ 4.49 × 10^3^	December April July September	0.1 M HCl	*Cystoseira* *schiffneri*	[54]
-	39.9 ~43 66.5 66.7	0.25 0.28 0.38 0.37	-	480.3 × 10^3^ 557.1 × 10^3^ ~357 × 10^3^ ~357 × 10^3^	-	Water 0.1 M HCl 5% Alcalase 5% Cellulase	*Sargassum* *angustifolium*	[55]
-	74.7 86.8 61.1	-	-	A-1.10 × 10^5^ PolyG-7.5 × 10^2^ PolyM-6.9 × 10^2^	May	0.1 M H_2_SO_4_	*Sargassum* *vulgare*	[57]

A—Alginate; PolyG—homopolymeric polyguluronate; PolyM—homopolymeric polymannuronate.

## Data Availability

The data presented in this study are available on request from the corresponding author.
